# Iron-Coordinated L-Lysine–Based Nanozymes with High Peroxidase-like Activity for Sensitive Hydrogen Peroxide and Glucose Detection

**DOI:** 10.3390/polym15143002

**Published:** 2023-07-10

**Authors:** Xiuqing Hou, Ruoxue Wang, Huijuan Zhang, Meng Zhang, Xiongwei Qu, Xiuli Hu

**Affiliations:** Institute of Polymer Science and Engineering, School of Chemical Engineering, Hebei University of Technology, Tianjin 300401, China; 13623269160@163.com (X.H.); w13513354867@163.com (R.W.); huijuan.zhang@foxmail.com (H.Z.); zhangmeng20220624@163.com (M.Z.)

**Keywords:** nanozymes, iron-coordinated L-lysine, precipitation polymerization, glucose, peroxidase

## Abstract

It is crucial to develop sensitive and accurate sensing strategies to detect H_2_O_2_ and glucose in biological systems. Herein, biocompatible iron-coordinated L-lysine–based hydrogen peroxide (H_2_O_2_)-mimetic enzymes (Lys-Fe-NPs) were prepared by precipitation polymerization in aqueous solution. The coordinated Fe^2+^ ion acted as centers of peroxidase-like enzymes of Lys-Fe-NPs, and the catalytic activity was evaluated via the oxidation of 3,3′,5,5′-tetramethylbenzidine (TMB) by H_2_O_2_. Therefore, a sensitive colorimetric detection sensor for H_2_O_2_ was constructed with a linear range of 1 to 200 μM and a detection limit of 0.51 μM. The same method could also be applied to highly sensitive and selective detection of glucose, with a linear range of 0.5 to 150 μM and a detection limit of 0.32 μM. In addition, an agarose-based hydrogel biosensor colorimetric was successfully implemented for visual assessment and quantitative detection of glucose. The design provided a novel platform for constructing stable and nonprotein enzyme mimics with lysine and showed great potential applications in biorelevant assays.

## 1. Introduction

Hydrogen peroxide (H_2_O_2_) and glucose play important roles in living organisms as a key intermediate in environmental and biological processes and as a messenger for cellular signal transduction [1,2]. Glucose is an indispensable substance in the body and a source of energy for cellular metabolism [3]. Human physiological diseases are closely related to the concentrations of glucose and H_2_O_2_, including diabetes, stroke, blindness, renal failure, peripheral neuropathy and other diseases [4,5,6]. Therefore, the accurate detection of H_2_O_2_ and glucose is essential in many fields, including food, pharmaceutical, clinical, industrial and environmental protection applications [7].

Peroxidase is usually used to catalyze the oxidation of H_2_O_2_, causing a color change in the substrate to determine the enzyme activity and the concentrations of substrates or H_2_O_2_ [8]. In living organisms, glucose is oxidized to glucuronic acid and H_2_O_2_ by the action of glucose oxidase (GOx) [9]. Therefore, the detection of glucose can be indirectly achieved through the detection of H_2_O_2_ under corresponding conditions. Enzymes are a class of organic molecules with catalytic functions produced by living cells, and peroxidases are natural enzymes with high catalytic efficiency, substrate specificity, and mild reaction conditions [10,11,12]. Since the chemical substance of enzymes is protein, they are prone to structural changes and inactivation in non-physiological environments, such as acids, bases and heat [13]. Coupled with the high cost of their preparation and purification processes, their applications are greatly limited [14,15,16,17]. While bionic enzymes have the advantages of low cost, good stability and easy storage [18], nanomaterials with enzymatic activities are increasingly becoming a popular research topic.

Most conventional nanozymes are metal-containing nanoparticles, such as Fe_3_O_4_, MnO_2_, V_2_O_5_, etc., and are biologically active, mainly due to the catalytic function of transition metals on compact nanoscale surfaces [19]. In nature, the binding pocket structure of biological enzymes provides a favorable spatial microenvironment in the vicinity of the active center, which is particularly important for high catalytic efficiency [20]. These features highlight the critical importance of active site components and supportive network environments for integration and stability. In 2007, a Fe_3_O_4_-based nanozyme was reported [21], which was also the earliest nanoparticle found to have peroxidase activity. Through research, it was found that the conversion between Fe^2+^/Fe^3+^ in a large number of iron atoms on the surface of the nanozyme is the key to ensure its enzyme activity. Therefore, iron-based nanozymes have attracted widespread attention due to their excellent catalytic activity, low cost, and good stability and have been widely applied in the field of biomedicine. Some iron-based nanomimetic enzymes have been used for glucose detection, such as MIL-53(Fe) [22], Fe-N-C SAzyme [23] and Fe/NC-800 [24]. However, the preparation of nanomaterials is labor-intensive, and most of them tend to aggregate during the preparation process with encapsulated catalytic sites and reduced specific surface area [25,26], which may affect their catalytic activity. Therefore, it remains challenging to explore new approaches to prepare novel nanozymes with a high density of active sites.

**Figure 1:**
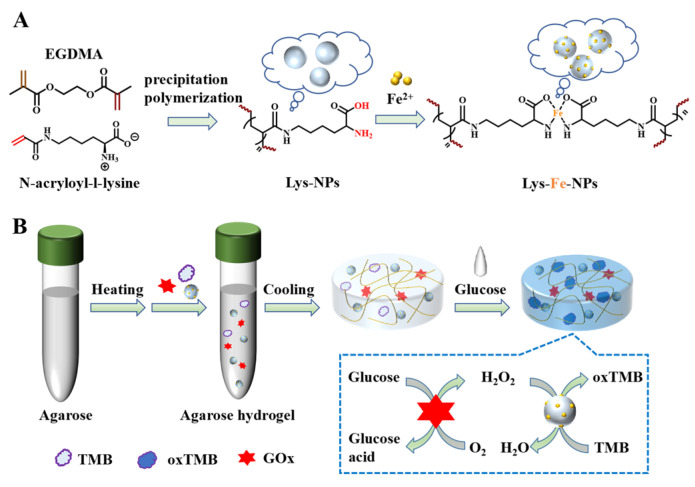
(**A**) Schematic diagram of the preparation of Lys-Fe-NPs. (**B**) Integration of agarose-based hydrogels for colorimetric detection of glucose.

Precipitation polymerization enables the efficient preparation of nanoparticles with precisely controlled size and uniform particle size distribution in a short period of time as well as the introduction of multiple functional groups, providing a novel and promising strategy for the preparation of monodisperse stable nanomaterials [27,28,29]. Herein, lysine-based nanoparticles, Lys-NPs, were prepared in one step by precipitation polymerization using N-acryloyl-l-lysine as the polymerizing monomer and ethylene glycol dimethacrylate (EGDMA) as the cross-linking agent. By controlling the contents of cross-linking agents, nanoparticles with different sizes and morphologies were obtained. Then, Fe^2+^ coordinates with the lysine chain in the nanoparticles (Figure 1A), and the introduction of Fe^2+^ imparts Lys-NPs peroxidase-like activity. Its activity was assessed by catalyzing the oxidation of 3,3′,5,5-tetramethylbenzidine (TMB) with H_2_O_2_ to produce a blue product. In addition, an agarose hydrogel glucose sensor was constructed (Figure 1B), which proved to provide rapid and easy visual assessment and quantitative detection of glucose.

## 2. Experiment

### 2.1. Materials

CuSO_4_•5H_2_O, NaOH, Na_2_CO_3_, 8-Hydroxyquinoline, ammonium persulfate (APS) and P-phthalic acid (TA) were bought from Macklin Co., Ltd. (Shanghai, China). Ethylene Glycol Dimethacrylate (EGDMA), L-Lysine hydrochloride, FeSO_4_•7H_2_O, TMB, H_2_O_2_, glucose, glucose oxidase (GOx), uric acid, galactose, fructose, L-cysteine, mannose, sucrose, chitosan, cysteine, glutathione, urea, Na_2_HPO_4_•12H_2_O, NaH_2_PO_4_•2H_2_O, acetic acid (HAc), sodium acetate (NaAc), etc., were purchased from Heowons, Chemistry Co., Ltd. (Tianjin, China). The HAc-NaAc buffer solution was prepared by HAc and NaAc, and we used Na_2_HPO_4_•12H_2_O and NaH_2_PO_4_-2H_2_O to prepare the phosphate buffer solution (PBS). All reagents purchased above were used directly without further treatment. Water used in the experiment was deionized water.

### 2.2. Preparation of N-Acryloyl-l-lysine

N-acryloyl-l-lysine was synthesized according to Ref [30]. Briefly, NaOH (3.66 g), Na_2_CO_3_ (4.73 g), L-Lysine hydrochloride (8.16 g) and CuSO_4_•H_2_O (5.58 g) were added to water to form a mixed solution. Subsequently, acryloyl chloride (5.0 g) was added dropwise to the mixed solution at 0 °C and stirred for 2 h at room temperature. The blue precipitate obtained by filtration was washed with water, ethanol and petroleum ether in turn, dried, and then added to a mixture of water and dichloromethane containing 8-hydroxyquinoline; the mixture was then stirred magnetically for 1 h. Purified by filtration and washing, the white solid obtained by freeze-drying was N-acryloyl-l-lysine.

### 2.3. Preparation of Lys-NPs and Lys-Fe-NPs

Amounts of 100 mg N-acryloyl-l-lysine, 40 mg EGDMA and 5 mg APS were added to the flask, increased to a temperature of 70 °C and reacted in an atmosphere of nitrogen for seven hours to produce a white precipitate; the resulting precipitate was centrifuged at 10,000 rpm for 5 min, then washed with ultrapure water and centrifuged, and the resulting product was dried in a freezer. Then, Lys-NPs (5 mg) and FeSO_4_•7H_2_O (5 mg) were added to the aqueous solution and reacted for 3 h. The precipitate was washed and centrifuged several times, and the yellow powder obtained after freeze-drying was Lys-Fe-NPs. The formulations for the preparation of Lys-Fe-NPs with different cross-linker contents are shown in Table 1.

### 2.4. Measurement of Peroxidase-like Activity of Lys-Fe-NPs

The activity assay was performed using a typical peroxidase substrate TMB [31] with the following four groups: (1) Lys-Fe-NPs (50 μL, 10 μg/mL) + TMB (100 μL, 2 mM) + H_2_O_2_ (100 μL, 2 mM); (2) TMB (100 μL, 2 mM) + H_2_O_2_ (100 μL, 2 mM); (3) Lys-Fe-NPs (50 μL, 10 μg/mL) + TMB (100 μL, 2 mM); (4) TMB (100 μL, 2 mM). Then, to each control group was added acetate buffer solution to a total volume of 650 μL. The solution was incubated at 40 °C for 5 min, and the absorbance from 400 to 750 nm was measured by a UV spectrophotometer. Similarly, UV absorption measurements of Lys-Fe-NPs with different cross-linker contents were performed in the presence of H_2_O_2_ (100 μL, 2 mM) and TMB (100 μL, 2 mM), respectively. The material concentration dependence of the catalysis activity was researched by measuring the concentration of Lys-Fe-NPs from 0–20 μg/mL. Additionally, the influence of the pH of solution was examined in different buffer solutions with pH changes from 2–10. Further, the temperature dependence of the nanoparticles in the temperature range of 25–80 °C and the catalytic activity at different substrate concentrations were also investigated.

### 2.5. Steady-State Kinetic Assay

Firstly, the TMB concentrations were kept constant (4 mM), and the H_2_O_2_ concentrations were changed from 0.1 mM to 4.0 mM. Sodium acetate buffer (400 μL, pH = 3.5) and Lys-Fe-NPs (50 μL, 10 μg/mL) were added to make different concentrations of mixed solutions and were incubated at 40 °C for 5 min; the absorbance of each solution was measured by UV-vis spectrophotometer [32]. Similarly, the concentrations of H_2_O_2_ were kept constant (4 mM), and the TMB concentrations (0.1–4.0 mM) were varied. The kinetic analysis with H_2_O_2_ as a substrate was studied. The absorbance was recorded at 652 nm every 60 s at ambient temperature. Then, the initial reaction rate was measured by the Beer-Lambert law [33]: c = A/(*ε* b), where c is the substrate concentration, A is the absorbance, ε is the molar absorption coefficient of the used colorimetric substrates (39,000 M^−1^ cm^−1^ at 652 nm for oxTMB) [34], and b is the thickness of the solution. The enzymatic parameters were determined by fitting the absorbance data to the following Michaelis–Menten equation [35]: 1/V = K_m_/V_max_ (1/[S] + 1/K_m_), where V is the initial reaction rate, V_max_ is the maximum reaction rate, and [S] is the substrate concentration. K_m_ is the Michaelis–Menten constant, which represents the affinity of the enzyme towards its substrate in the catalytic reaction.

### 2.6. Experimental Method for the Measurement of H_2_O_2_ and Glucose

First, for experiments to detect H_2_O_2_, 400 μL of HAc-NaAc buffer solution (pH = 3.5), 50 μL of Lys-Fe-NPs solution (10 μg/mL), 100 μL of TMB solution (2 mM) and 100 μL of different concentrations of H_2_O_2_ solution (1–1000 μM) were added sequentially to the centrifuge tubes and incubated in a shaker at 40 °C for 5 min before measuring the absorbance of the reaction solution in a UV-Vis spectrophotometer. The corresponding UV-Vis spectra were recorded between 400 and 750 nm, and the standard curves were plotted and measured three times in parallel. In order to determine the selectivity of H_2_O_2_ detection, comparison experiments were carried out by replacing H_2_O_2_ with TMB and common metal ions, and the final concentrations were set as 1 mM [36].

Glucose assay was performed as follows: 100 μL of 1 mg/mL glucose oxidase aqueous solution and 100 μL of different concentrations (0.5–400 μM) of glucose PBS solutions (pH = 6.4) were mixed well and reacted for 50 min at 37 °C. Then, 400 μL of sodium acetate buffer (pH = 3.5), 50 μL of Lys-Fe-NPs (10 μg/mL) and 100 μL of TMB solution (4 mM) were added to the above mixture and incubated in a shaker at 37 °C for another 50 min. Finally, the corresponding UV-Vis spectra were recorded between 400 and 750 nm, and the standard curves were plotted and measured three times in parallel. To test the selectivity of the glucose assay [37], a control experiment was performed in which lactose, fructose, sucrose, urea, cysteine, homocysteine, bovine serum albumin (BSA) and L-glutathione (GSH) were used instead of glucose, and the final concentrations were set as 4 mM.

### 2.7. Preparation of Integrated Agarose-Based Hydrogel Film

Agarose (0.05 g) was dissolved in 2.4 mL of ultrapure water and heated until the agarose was totally dissolved. Lys-Fe-NPs1 (300 μL, 50 μg/mL), TMB (100 μL, 4 mM), NaAc-HAc buffer solution (200 μL, pH 3.5) and glucose oxidase solution in PBS (100 μL, 1 mg/mL, pH = 6.4) were added. Then, the mixture was stirred until uniformly mixed. The gel film was naturally cooled, and 100 μL of different concentrations of glucose solutions (0.2–3 mM) were added dropwise; the relative UV-Vis spectra were measured between 400 and 750 nm, and the standard curves were plotted.

## 3. Results and Discussion

### 3.1. Preparation of Lys-Fe-NPs

**Figure 2:**
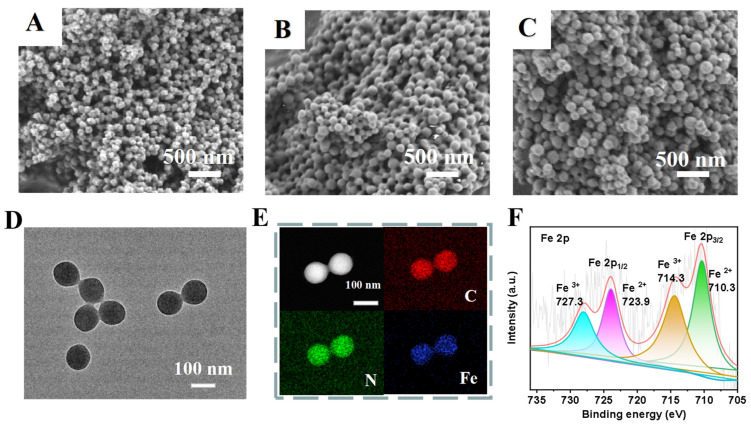
SEM images of Lys-Fe-NPs with different counts of cross-linker: (**A**) Lys-Fe-NPs1, (**B**) Lys-Fe-NPs2 and (**C**) Lys-Fe-NPs3. (**D**) TEM image and (**E**) EDS mapping image of Lys-Fe-NPs1. (**F**) Fe 2p XPS spectra of Lys-Fe-NPs1.

Firstly, Lys-NPs were prepared in one step by precipitation polymerization (Figure S1) using N-acryloyl-l-lysine (Figure S2) as the polymerizing monomer and EGDMA as the cross-linking agent (Table 1). Homogeneous and dispersed spherical nanoparticles were obtained after complexation with iron, as shown in SEM images in Figure 2A–C. The size of the nanoparticles could be controlled by adjusting the amount of cross-linker EGDMA, as shown in Table 1. As the contents of the cross-linker increase, the nanoparticle size increases. The results of dynamic light scattering (DLS) showed that the diameter was slightly larger compared to that determined by SEM (Table 1), which could be accounted for by the presence of a hydrated layer of nanoparticles. Lys-NPs and their iron ion complexes (Lys-Fe-NPs) have similar particle size and size distribution (Figure S3). The energy dispersive X-ray spectroscopy (EDS) mapping images of Lys-Fe-NPs1 confirm homogeneous distributions of carbon, nitrogen and iron elements, confirming the successful complexation of iron ions with Lys-NPs (Figure 2D,E). In the FT-IR spectra, the appearance of characteristic peaks corresponding to O-Fe bonds at 602 cm^−1^ and the weakening of the peak at 3428.9 cm^−1^ further indicate the successful coordination of iron ions [30] (Figure S4). Then, the stability of the three Fe^2+^-complexed Lys-Fe-NPs with different contents of cross-linking agent were evaluated by detecting their size and size distribution at room temperature in PBS solution; all the nanoparticles maintained stable and monodispersed in PBS solution for as long as 7 days, verifying their superior stability and insuring the feasibility of long-term storage (Figure S5). In addition, the chemical constituents and valency states were analyzed by XPS. Figure S6 obviously showed distribution of the four typical peaks of C 1s, N 1s, O 1s and Fe 2p in Lys-Fe-NPs1, with atomic contents of 67.58%, 6.23%, 23.85% and 2.34%, respectively. Due to the ligand field and spin-orbit splittings, the peaks at 727.3 eV and 723.9 eV belong to Fe^3+^2p1/2 and Fe^2+^2p1/2, and binding energy peaks of Fe^3+^2p3/2 and Fe^2+^2p3/2 were observed at 714.3 eV, and 710.3 eV, respectively (Figure 2F) [38]. The EDS spectrum shown in Figure S7 recently confirmed the presence of C (67.69%), N (5.22%), N (24.55%) and Fe (2.54%) in Lys-Fe-NPs1, consistent with the XPS result. All the above results suggest the successful preparation of Lys-Fe-NPs1. Considering that the monodispersity of the three nanoparticles is similar, and the size of Lys-Fe-NPs1 is the smallest, Lys-Fe-NPs1 was used mainly for the following experiments.

### 3.2. Peroxidase-like Catalytic Activity of Lys-Fe-NPs

**Figure 3:**
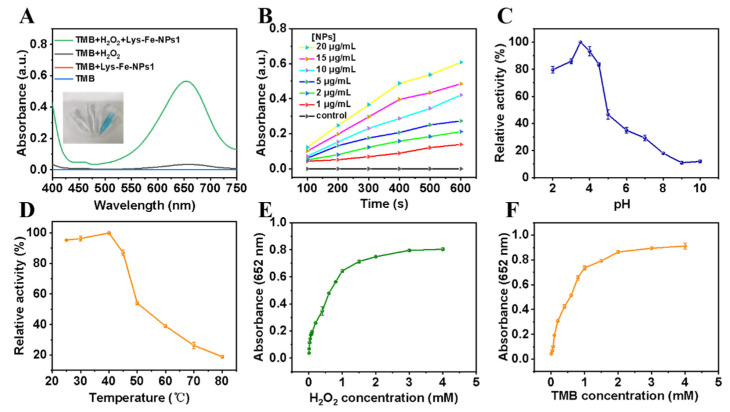
(**A**) UV-vis absorption spectra of solutions with different compositions; inset: corresponding pictures of the different solution samples. (**B**) Absorbance of the TMB-H_2_O_2_ reaction system catalyzed by different concentrations of Lys-Fe-NPs1 depending on time. (**C**–**F**) Effect of various factors on the peroxide-like properties of Lys-Fe-NPs1: (**C**) pH of solutions, (**D**) temperature of reaction, (**E**) concentration of H_2_O_2_, (**F**) concentration of TMB.

Owing to the presence of iron, Lys-Fe-NPs may have a catalytic role in simulating the peroxidase enzymatic activity. By catalyzing TMB in the presence of H_2_O_2_ to assess the peroxidase-like activity of Lys-Fe-NPs, when Lys-Fe-NPs1 was added to the TMB-H_2_O_2_ solution, the solution gradually became blue and had a noticeable UV characteristic absorption peak at 652 nm, which was considered as the generation of oxTMB. In contrast, in the absence of Lys-Fe-NPs1, the absorption in the wavelength range of 400–750 nm was negligible (Figure 3A). It was found that Lys-Fe-NPs2 and Lys-Fe-NPs3 showed similar peroxidase activity under the same experimental conditions by TMB UV absorption assay (Figure S8). All the above results indicate that Lys-Fe-NPs can oxidize TMB in the presence of H_2_O_2_ to turn the solution blue and have a maximum absorption at 652 nm, with good peroxidase-like activity.

The plot of absorbance at 652 nm depending on time (Figure 3B) indicated that as the concentrations of Lys-Fe-NPs1 increased, the absorbance changed and the reaction rate increased significantly. Common factors influencing enzyme activity, including the pH of the solution, the temperature during catalytic reaction, and the concentrations of substrate were then measured. The catalytic activity of peroxidase was compared for Lys-Fe-NPs1 at pH values from 2 to 10 (Figure 3C) and at different temperatures from 25 to 80 °C (Figure 3D). It was proven experimentally that the solution pH for the nanoparticle Lys-Fe-NPs1 to reach the optimal catalytic activity is 3.5 with a reaction temperature of 40 °C. The reason is that the weak acid reaction system facilitates the formation of TMB oxidation intermediates, H_2_O_2_ decomposes by its own oxidation when the temperature exceeds 40 °C, and the resulting TMB oxidation intermediate is unstable, so the catalytic activity gradually decreases. Therefore, we chose pH 3.5 and 40 °C as the standard conditions for the subsequent experiments. Subsequently, we examined the effects of different substrates as well as different substrate concentrations on the catalytic activity of Lys-Fe-NPs1, and the results obviously showed that the absorbance value at 652 nm grew gradually with increasing H_2_O_2_ or TMB concentrations (Figure 3E,F). Furthermore, no inhibition of the reaction catalyzed by Lys-Fe-NPs1 was found at H_2_O_2_ concentrations up to 4.0 mM or TMB concentrations up to 4.0 mM, indicating that Lys-Fe-NPs1 also exhibited stable catalytic activity in high concentrations of H_2_O_2_ or TMB.

### 3.3. Kinetic Data and Reaction Mechanism of Lys-Fe-NPs

**Figure 4:**
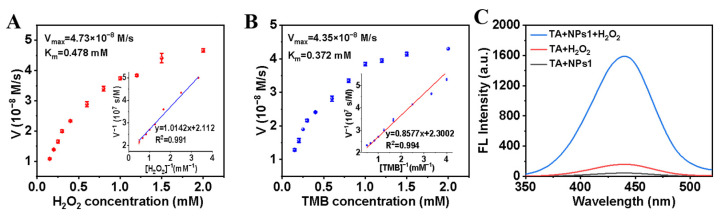
Kinetic assay of Lys-Fe-NPs1: (**A**) 4 mM TMB with varied concentrations of H_2_O_2_; inset: Lineweaver–Burk plots. (**B**) 4 mM H_2_O_2_ with varied concentrations of TMB; inset: Lineweaver–Burk plots. (**C**) Fluorescence spectra of TA + H_2_O_2_ + Lys-Fe-NPs1, TA + H_2_O_2_ and TA + Lys-Fe-NPs1 after incubation.

The measurement of catalytic activity and kinetics of the peroxidase-like Lys-Fe-NPs using standardized assays was performed according to the research. Based on the above catalytic activity–dependent substrate concentration analysis, we investigated the kinetic parameters of the enzymatic reaction of Lys-Fe-NPs1 using homeostatic kinetics. A typical Michaelis–Menten curve was obtained for Lys-Fe-NPs1 in a suitable concentration range of H_2_O_2_ and TMB, and Km was determined as a measure of the affinity of the enzyme for the substrate. A low Km value represents a strong affinity, and vice versa. In the Lys-Fe-NPs1/TMB/H_2_O_2_ system, other conditions were kept unchanged, except the amount of H_2_O_2_. It was found that K_m_ = 0.478 mM and V_max_ = 4.73 × 10^−8^ M/s of H_2_O_2_ (Figure 4A). Other conditions were kept unchanged, but the amount of TMB was varied. As shown in Figure 4B, K_m_ = 0.372 mM and V_max_ = 4.35 × 10^−8^ M/s of TMB. This indicated that Lys-Fe-NPs1 had a high affinity for both H_2_O_2_ and TMB.

From Figure 4A,B, it can be observed that the trends of the initial rates of TMB and H_2_O_2_ with concentrations are linear and parallel, indicating that the catalytic process of Lys-Fe-NPs1 is also consistent with the ping-pong mechanism [39]. This means that Lys-Fe-NPs1 first bind and react with the first substrate and then release the products of the first reaction before reacting with the second substrate.

The catalytic oxidation of TMB may be due to the hydroxyl radical (•OH) that is generated by the peroxidase-assisted H_2_O_2_ decomposition [23]. We adopted typical hydroxyl radical trapping agent TA to detect the generation of hydroxyl radicals. The non-fluorescent TA combines with •OH to produce the highly fluorescent 2-hydroxyterephthalic acid, with a distinct peak at 440 nm. As shown in Figure 4C, the mixed solutions of TA and Lys-Fe-NPs1 showed no significant intensity of 2-hydroxyterephthalic acid, and mixed solutions containing TA and H_2_O_2_ appeared faintly fluorescent at 440 nm. Only in the presence of both Lys-Fe-NPs1 and H_2_O_2_ was intense fluorescence found at 440 nm. The results suggest that more •OH was produced in the system of [TA + H_2_O_2_ + Lys-Fe-NPs1]. H_2_O_2_ and Lys-Fe-NPs1 were two important factors for the formation of 2-hydroxyterephthalic acid. It has further been shown that Lys-Fe-NPs1 promotes the decomposition of H_2_O_2_ to form more •OH, while TMB binds to •OH to produce oxTMB.

### 3.4. Colorimetric Assays for H_2_O_2_ and Glucose

**Figure 5:**
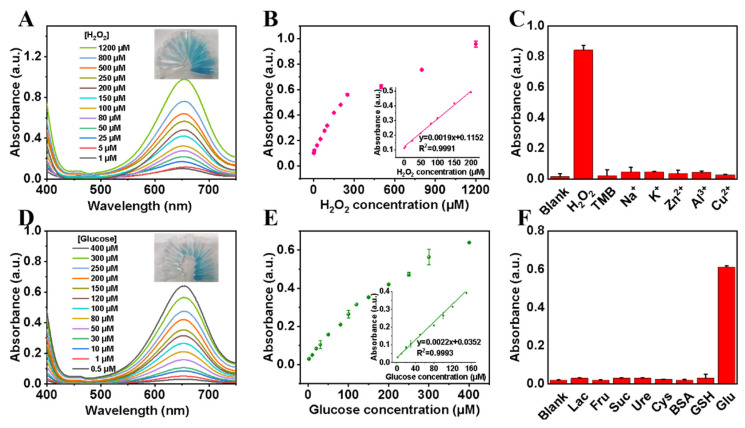
(**A**) UV-vis absorption spectra of Lys-Fe-NPs1 in different H_2_O_2_ concentrations. (**B**) Absorbance of oxTMB for Lys-Fe-NPs1 at different H_2_O_2_ concentrations; inset: standard curve (at 652 nm). (**C**) The selectivity of Lys-Fe-NPs1 for H_2_O_2_ detection. (**D**) UV-vis absorption spectra of Lys-Fe-NPs1 in different glucose concentrations. (**E**) Absorbance of oxTMB for Lys-Fe-NPs1 at different glucose concentrations; inset: standard curve (at 652 nm). (**F**) Experiment on the specific selectivity of Lys-Fe-NPs1 for glucose detection.

Since the color change of oxTMB created by Lys-Fe-NPs1 was related to the concentration of H_2_O_2_, a colorimetric method for the assay of H_2_O_2_ was developed, with optimized conditions. In the Lys-Fe-NPs1/TMB/H_2_O_2_ reaction system, the concentrations of TMB and Lys-Fe-NPs1 were kept at 2 mM and 10 μg/mL, respectively. As the final H_2_O_2_ concentrations changed from 1 to 1000 μM, The color of the measured solution samples became darker, which revealed the creation of blue oxTMB (Figure 5A). The different absorbance values corresponding to different H_2_O_2_ concentrations were useful for quantification (Figure 5B), which can further illustrate the correspondence between the concentration of H_2_O_2_ and the absorbance of oxTMB. The H_2_O_2_ concentration response equation was y = 0.0019x + 0.1152; this equation had a good linear relationship (R^2^ = 0.9991). The linear range was from 1 to 200 μM, and the detection limit (LOD) of H_2_O_2_ was 0. 51 μM (S/N = 3).

In addition, the selectivity of Lys-Fe-NPs1 was divided into selectivity for H_2_O_2_ and selectivity for glucose by investigating the response of the Lys-Fe-NPs1-GOx-TMB system to common interfering chemicals. Compared with common metal ions and TMB at the same substrate concentration, only H_2_O_2_ caused the reaction of Lys-Fe-NPs1 (Figure 5C).

Glucose testing has raised concerns because glucose is a key indicator in the treatment of diabetes in a clinical setting. It is commonly known that in the presence of GOx, glucose is oxidized to form gluconic acid and H_2_O_2_; thus, the H_2_O_2_ concentration is linearly related to the glucose concentration. Hence, when these two different catalytic reactions are correlated in the presence of GOx and Lys-Fe-NPs1 hybrid nanomaterials, the oxTMB concentration should also be linearly related to the glucose concentration. In the Lys-Fe-NPs1-GOx-TMB reaction system, As the glucose concentration increased from 0.5 to 400 μM, the color of the examined samples became deeper (Figure 5D), which indicated the creation of blue oxTMB. The different absorbance values corresponding to different glucose concentrations were useful for quantification, which can further illustrate the correspondence between the concentration of glucose and the absorbance of oxTMB (Figure 5E). The results showed that the glucose concentration response equation was y = 0.0022x + 0.0352; this equation had a good linear relationship (R^2^ = 0.9993). The linear range was from 0.5 to 150 μM, and the limit of detection (LOD) of glucose was 0.32 μM (S/N = 3).

To test the selectivity of this colorimetric method, a number of possible interferents, such as lactose, fructose, sucrose, urea, cysteine and homocysteine, were used in a control experiment under the same conditions. As illustrated in Figure 5F, only glucose produced a significant color change in the solution, while the interfering chemicals mentioned above remained colorless and identical to the blank, which strongly indicates the high selectivity of Lys-Fe-NPs for glucose. Furthermore, the absorbance in the presence of the interfering molecule was essentially the same as that of glucose alone (Figure S9), which further evidenced the selectivity of the system. Therefore, the colorimetric method allows a highly selective detection of glucose, which is attributed to the specificity of GOx for glucose.

### 3.5. Lys-Fe-NPs-GOx-TMB Agarose-Based Hydrogel

To investigate the biological utility of the Lys-Fe-NPs-GOx-TMB colorimetric sensing system, especially for medical detection, we designed a low-cost agarose hydrogel biosensing medium containing GOx, TMB, and Lys-Fe-NPs1 for sensitive detection of glucose.

**Figure 6:**
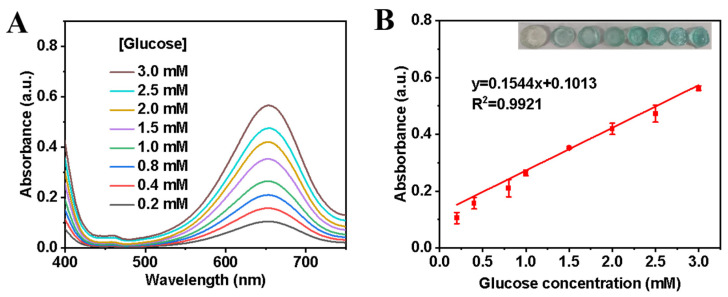
(**A**) UV-vis absorption spectra of Lys-Fe-NPs in different glucose concentrations. (**B**) Linear calibration curve of the absorbance at 652 nm against glucose concentrations. Inset: Agarose-based hydrogels with different glucose concentrations.

Then, we dropped different concentrations of glucose on the above hydrogel film to analyze the absorption spectra and the corresponding color changes. As demonstrated in Figure 6A,B, the hydrogel film gradually changed from colorless to blue with increasing glucose concentration from 0.2 to 3 mM, while the absorbance at 652 nm also increased. A good linear correlation was also obtained in the range of 0.2–3 mM (Figure 6B). Sensitive sensing of glucose may be attributed to multiple active sites on the structural surface of Lys-Fe-NPs1. Considering that glucose concentrations in the blood of diabetic patients and healthy individuals are 9–40 mM and 3–8 mM, respectively [40], the colorimetric sensor developed for the determination of glucose is sufficiently sensitive.

## 4. Conclusions

In summary, we prepared iron-coordinated lysine-based nanozymes by precipitation polymerization with homogeneous size, stability and high peroxidase-like catalytic activity, which can sensitively detect H_2_O_2_ and glucose with low detection limits of 0.51 μM and 0.32 μM, respectively. After the accurate colorimetric detection of glucose in solution by the Lys-Fe-NPs-GOx-TMB system, we developed an agarose-based hydrogel colorimetric biosensor for the successful visualization and quantitative assessment of glucose. The prepared colorimetric method for detection of H_2_O_2_ and glucose using Lys-Fe-NPs has good sensitivity and great potential applications in biological detection fields.

## Figures and Tables

**Table 1:** The DLS data of Lys-Fe-NPs with different cross-linker (EGDMA) contents.

Sample	Cross-Linker Content (wt%)	Diameter ^a^ (nm)	Diameter ^b^ (nm)	PDI ^b^
Lys-NPs	40	93.7	108.4	0.193
Lys-Fe-NPs1	40	98.6	122.3	0.201
Lys-Fe-NPs2	50	150.1	178.9	0.132
Lys-Fe-NPs3	60	173.4	202.4	0.117

^a^ Particle size determined by SEM, ^b^ Hydrodynamic diameter and PDI measured by DLS in PBS 7.4 at 25 °C.

## Data Availability

Not applicable.

## Supplementary Materials

The following supporting information can be downloaded at: https://www.mdpi.com/article/10.3390/polym15143002/s1.
