# Supplementary material for: Iron-Coordinated L-Lysine–Based Nanozymes with High Peroxidase-like Activity for Sensitive Hydrogen Peroxide and Glucose Detection
Source: Polymers (Basel). 2023 Jul 10;15(14):3002. doi: 10.3390/polym15143002 (PMC10383789; doi:10.3390/polym15143002)
Supplement: Supplementary file 1 [file polymers-15-03002-s001.zip › polymers-2485378-supplementary.pdf]

Supporting Information

S1.Material characterization methods

S1.1 Fourier Transform Infrared Spectroscopy Measurement (FT-IR)

Fourier transform infrared spectroscopy can analyze the functional groups contained in a substance, and thus obtain information on the chemical composition and chemical bonding of the substance. The infrared spectra of samples Lys-NPs and Lys-Fe-NPs were analyzed by Fourier infrared spectroscopy for the following tests: Firstly, an appropriate amount of dried potassium bromide was taken and ground into powder with a mortar and pestle, and then a small amount of the sample was added and continued to be ground until the sample and the potassium bromide powder were completely mixed well.The mixed powder was added to a mold and pressed into transparent potassium bromide tablets. Put the potassium bromide tablets into the FTIR spectrometer and scan the potassium bromide tablets containing the sample in the range of 4000 cm^{-1} ~ 500 cm^{-1}.

S1.2 Scanning electron microscope observation (SEM)

Scanning electron microscopy observation of the surface morphology of nanoparticles Lys-Fe-NPs was tested as follows: the lyophilized sample powder Lys-Fe-NPs was evenly spread on a conductive silicon wafer and placed in a field emission scanning electron microscope to observe the surface morphology.

S1.3 X-ray photoelectron spectroscopy (XPS)

X-ray photoelectron spectroscopy is a commonly used surface analysis technique to determine the chemical composition, valence, molecular structure and other information of the material surface.The nanoparticles were spread uniformly on tin foil and their elements of Fe, N, C and O were quantified.

S1.4 Transmission Electron Microscope (TEM)

The elemental distribution of nanoparticle Lys-Fe-NPs1 was observed by transmission electron microscopy as follows: aqueous solution of well-dispersed Lys-Fe-NPs1 was added dropwise onto a copper grid and placed in a field emission transmission electron microscope to observe its elemental distribution and determine its relative content.

S1.5 Dynamic Light Scattering (DLS)

The distribution of nanoparticles was measured for a nanoparticle PBS solution at a concentration of 1 mg/ml, tested three times in parallel, and measured for seven consecutive days.

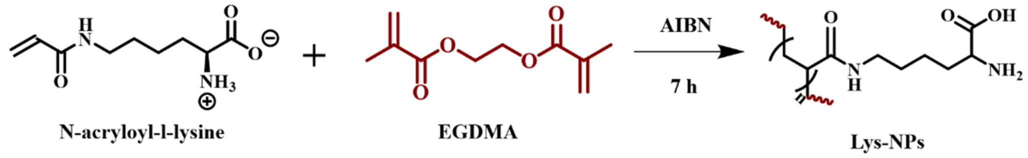

Figure S1. Preparation flow chart of Lys-NPs.

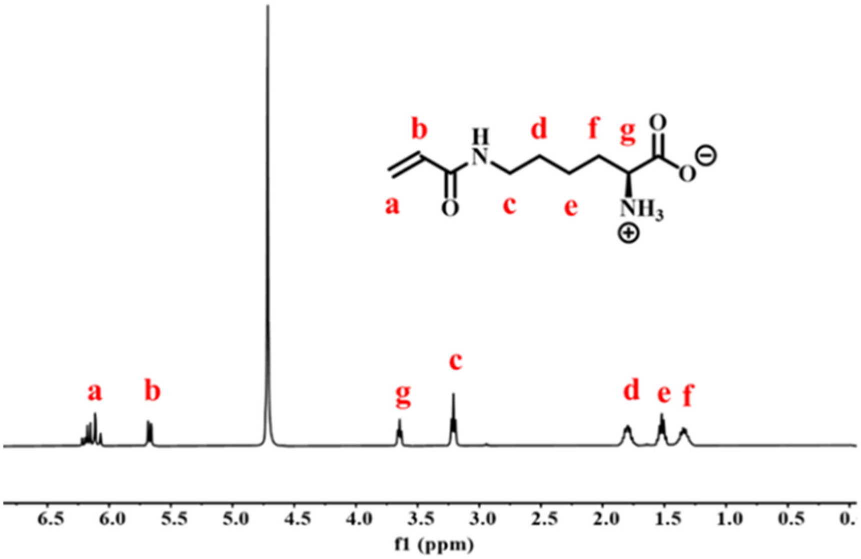

Figure S2. ^{1}H NMR spectrum of N-acryloyl-l-lysine.

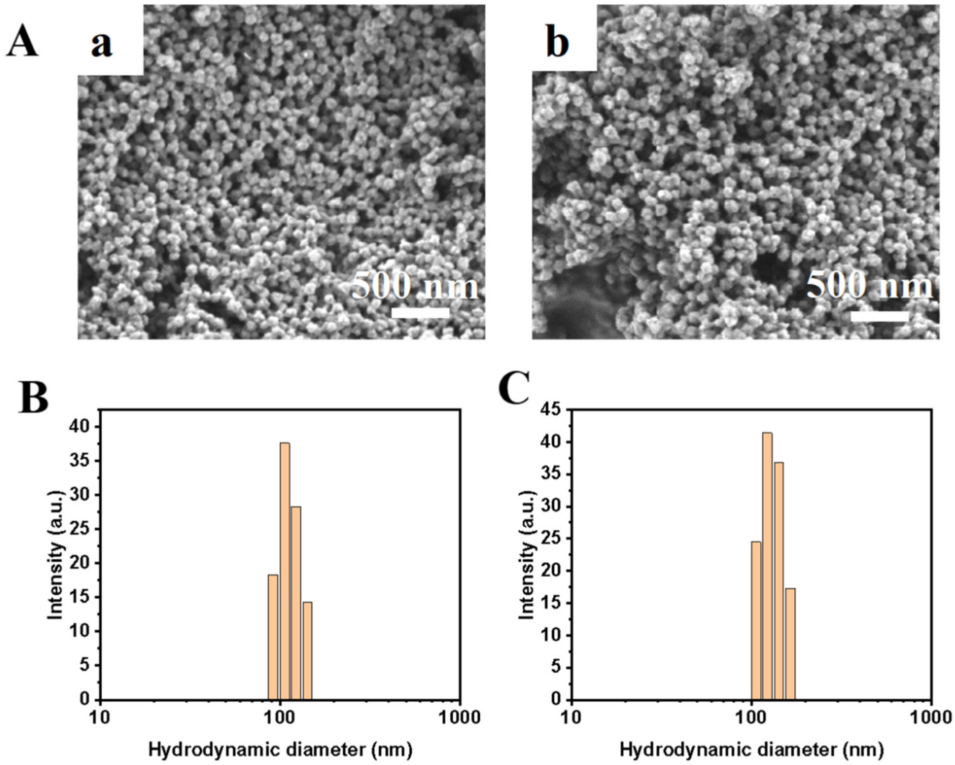

Figure S3. (A)SEM images of (a)Lys-NPs; (b)Lys-Fe-NPs1;.(B)DLS of Lys-NPs; (C)DLS of Lys-Fe-NPs1.

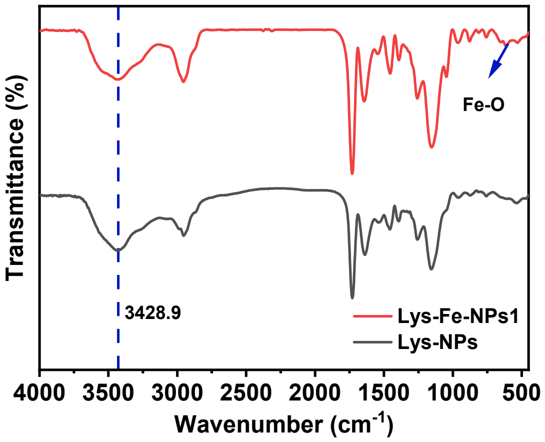

Figure S4. (e) FT-IR spectra of Lys-NPs and Lys-Fe-NPs1.

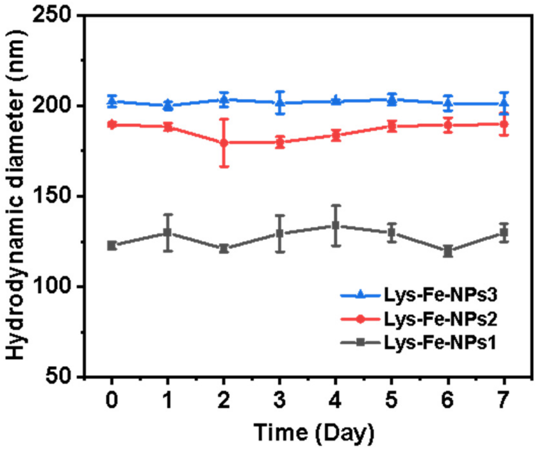

Figure S5. The DLS of Lys-Fe-NPs1, Lys-Fe-NPs2 Lys-Fe-NPs3 for 7 days.

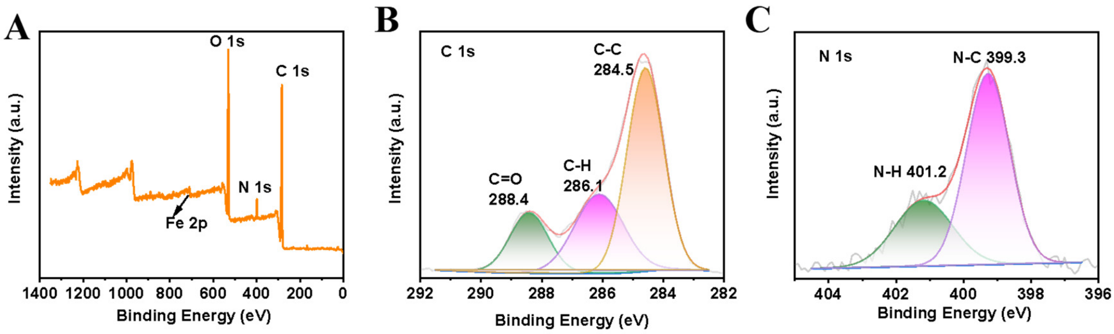

Figure S6. The XPS survey spectrum of (A) Full Spectrum, (B)C 1s, and (C) N 1s in Lys-Fe-NPs1.

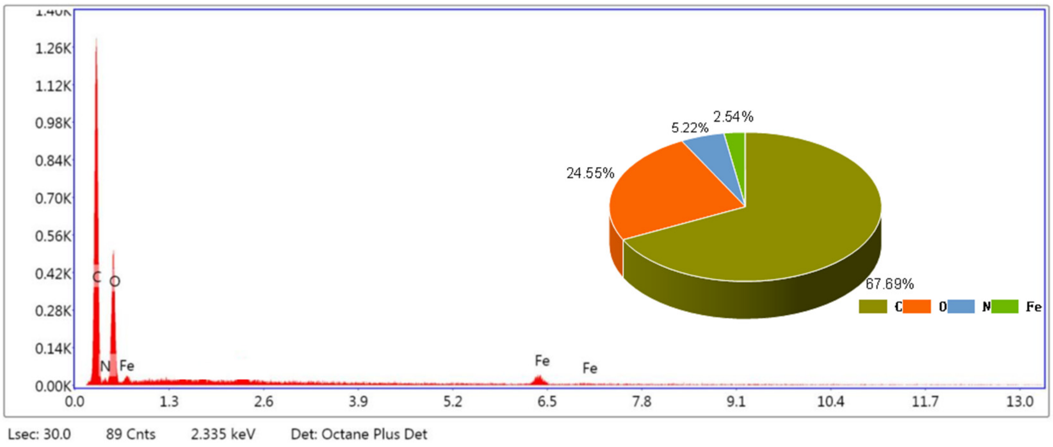

Figure S7. Energy dispersive X-ray analysis (EDS) spectra and element atomic percentage of Lys-Fe-NPs1.

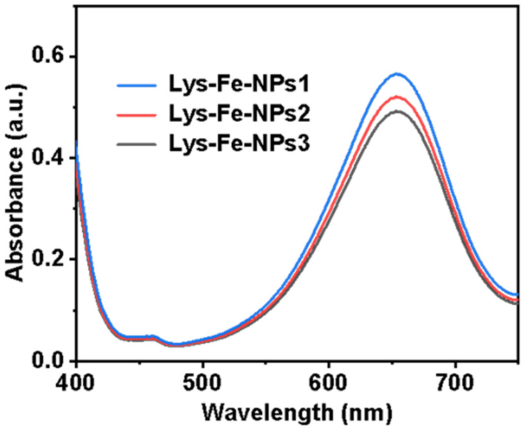

Figure S8. The peroxidase-like activity of Lys-Fe-NPs1, Lys-Fe-NPs2 and Lys-Fe-NPs3.

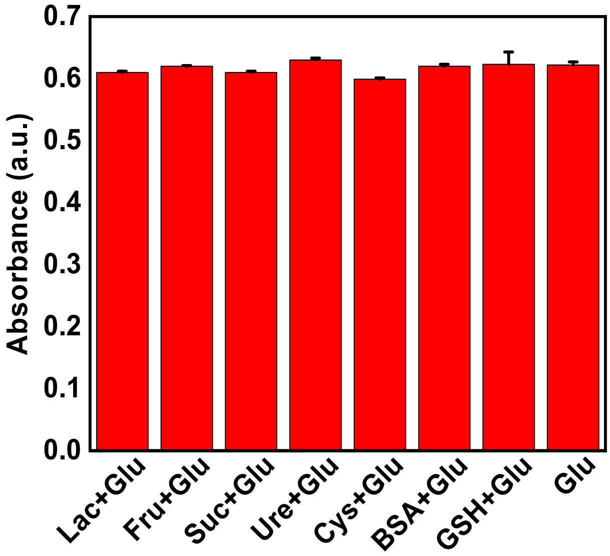

Figure S9. The competition experiment of glucose with the coexisting substances was detected in solution containing glucose and one of disrupters.
